# IL-1β, TNF-α, and IL-10 reduce cell viability and differentially alter biofilm structure and gene expression levels in *Staphylococcus aureus* USA 300

**DOI:** 10.3389/fimmu.2025.1665397

**Published:** 2025-12-05

**Authors:** Adriana N. Zavala-Hernández, Christian Salto-Reyes, Alejandro Bravo-Patiño, Víctor M. Baizabal-Aguirre, Juan J. Valdez-Alarcón

**Affiliations:** Centro Multidisciplinario de Estudios en Biotecnología, Facultad de Medicina Veterinaria y Zootecnia, Universidad Michoacana de San Nicolás de Hidalgo, Tarímbaro, Michoacán, Mexico

**Keywords:** *Staphylococcus aureus*, biofilm, cytokines, microbial endocrinology, gene expression

During infections, *S. aureus* is exposed to signal molecules from its host, including hormones, cytokines, and chemokines of the immune system, as well as signals from the microbiota that share the same human niches. *S. aureus* infections cause inflammation and immune modulation signals induction that may be perceived by bacterial biofilms attached to infected tissues. *S. aureus* is known to respond to catecholamines, steroid hormones, defensins and other host signals. In this work, we analysed whether *S. aureus* was capable of responding to immunomodulatory signals, such as TNFα, IL-1. (proinflammatory), or IL-10 (anti-inflammatory) cytokines. Biofilm formation and structure, as well as the relative gene expression of global virulence regulators, were evaluated *in vitro* in *S. aureus* strain USA300, of human origin. All cytokines decreased the biofillm formation index (BFI) value in a dose-dependent manner at concentrations ranging from 0.1 ng/ml to 100 ng/ml. Cytokines exhibit an inhibitory effect on cell viability and induce cytokine-specific structural changes in biofilms, as well as specific alterations in the expression of global regulators’ genes. These suggest specific detection and response pathways in *S. aureus* to each cytokine. 

## 1 Introduction

Despite all the knowledge generated to date on pathogenesis mechanisms, there is still a long way to go to control *S. aureus* infections. *S. aureus* is a versatile pathogen that causes Community-acquired (impetigo, toxic shock syndrome, skin and soft tissue infections such as abscesses, boils, and cellulitis, mastitis), Hospital- or healthcare-acquired (surgery associated infections, pneumonia, bacteraemia, osteomyelitis, endocarditis) and Livestock- and companion animal-associated infections (mastitis, pyoderma, surgery associated infections) which may be of zoonotic origin (1–3) When *S. aureus* initiates an infection, the host’s innate immune system responds, triggering an inflammatory process. This inflammation is promoted by the presence of lymphocytes, which release pro- and anti-inflammatory cytokines. *S. aureus* pathogenesis is closely related to the inflammatory response. The strategies of *S. aureus* to evade the immune system can be divided into five groups: a) Inhibition of the recruitment of neutrophils; b) Inhibition of phagocytosis; c) Inhibition of destruction by reactive oxygen species (ROS); d) Destruction of neutrophils and e) Resistance to antimicrobial peptides (2). To survive and adapt within the host, *S. aureus* has evolved a regulatory network to control the production of virulence factors. The regulatory network and virulence factors are known as accessory genes, as they are not essential for normal, vegetative growth (4). Detection of cell density (*quorum* sensing, QS) is a requirement for the expression of various virulence factors. Cell-to-cell communication by QS allows the bacterium to activate or repress specific genes by secreting specific molecules called autoinducers (AI). In Gram-negative bacteria, AI-1 has a role in intracellular communication, AI-2 is associated with interspecies communication, and AI-3 is inhibited by adrenergic receptor antagonists, suggesting that this molecule is similar in structure to epinephrine and norepinephrine (5). In Gram-positive bacteria, one of the cell density detection systems is the Agr system. It was first described in 1986 and is considered a global regulator of virulence. This is one of the several two-component systems (TCS) in *S. aureus* genomes. Most *S. aureus* strains encode sixteen different TCS, including WalKS, which is essential, and others such as AgrAC, SaeRS, and ArlRS associated with virulence, which regulate secreted proteins that affect the host (4). At high cell density, *S. aureus* AgrB transmembrane protein processes the intercellular AgrD precursor peptide and exports the mature autoinducing peptide (AIP) to the extracellular environment, which in turn stimulates the AgrC histidine-kinase receptor that, when activated, downstream phosphorylates AgrA transcription factor. RNAIII is induced by phosphorylated AgrA for the control of group behaviour (6). Cytoplasmic proteins regulating gene expression are also found; examples are the family of transcriptional regulators, SarA, and the sigma factors (SigB and SigH) (4), that participate in the regulatory network of biofilm formation.

The formation of bacterial biofilms is a process described in five main stages: (I) adhesion or reversible binding, (II) irreversible binding and multiplication, (III) exodus, (IV) biofilm maturation, (V) dispersal or detachment phase (7). The formation of biofilms favours the persistence and resistance of bacteria to hostile environments, such as those found in food processing, and helps them tolerate stress conditions as defence mechanisms of the host or as part of the innate immune response. Biofilms also limit the diffusion of small molecules as antibiotics, rendering the bacteria tolerant or resistant. In *S. aureus* biofilms, bacterial cells are embedded in a polymeric extracellular matrix (EPS), composed of intercellular adhesion polysaccharides, proteins, extracellular DNA, and RNA (7, 8). Biofilm matrix synthesis is related to the ability to survive in diverse environmental conditions and to evade the host’s innate immune response. A bacterial biofilm may be composed of a single kind of microorganism or a community of bacteria, fungi, archaea, protozoa, and yeasts. It presents channels in its structure that control the release of gases, nutrient flux, and antimicrobials access (9, 10). Previously, Ibarra et al. (11) identified 135 transcription factors in *S. aureus* USA-300 strain. Biofilm formation is known to be mainly regulated by transcription factors SaeR, LytSR, CodY, MgrA, SarA, Rot, SigB, and the *quorum* sensing system Agr, among others (12).

Microbial endocrinology, as an emerging discipline, studies gene expression of pathogenic bacteria in response to host immune, nervous, and physiological host signaling (4, 13, 14). It has been reported that *S. aureus* is capable of detecting and responding to host signals such as catecholamines (epinephrine, norepinephrine, dopamine), steroid hormones (estrogens, progesterone, testosterone), α-defensins (HNP-1), short-chain fatty acids, cholecalciferol, which alter several responses in *S. aureus*, such as biofilm formation, growth rate, cell permeability, SaeRS´s (a global regulator of exoprotein gene expression) activation, internalization ability on phagocytic cells, among others (15–20).

*S. aureus* activates the inflammasome, subsequently inducing IL-1β and activating NF- κB transcription factor, which is a component of TNFα-induced gene expression (21). In atopic dermatitis, *S. aureus* also induces IL-10, an anti-inflammatory cytokine in Th2 cells (22). Oviedo-Boyso and colleagues reported that IL-1β and TNFα, when added to bovine endothelial cells before infection with *S. aureus*, increase their ability to eliminate intracellular *S. aureus* and *S. epidermidis* (23), thus remarking the role of IL-1β and TNFα in the interaction. An *S. aureus–*host cell communication may be established from the onset of infection. It is possible that, as early-response signals, cytokines involved in the *S. aureus*–induced inflammation process, such as IL-1β, TNFα (proinflammatory), and IL-10 (anti-inflammatory), may also be sensed by *S. aureus* cells. To test this, we chose biofilm formation as an early virulence feature to evaluate the possible effect of cytokines. In this work, we evaluate bacterial cell viability, biofilm formation capacity, biofilm structure, and gene expression of global regulators of biofilm formation (*agrA*, *sarA*, *saeR*, *rnaIII*, and *sigB*) to show the effect of the above-mentioned cytokines on *S. aureus* USA 300, a reference strain from community-acquired, human soft tissue infections. We also evaluate some aspects of cytokine response in a strain associated with bovine mastitis (ATCC 27543). These strains share similar genetic backgrounds.

## 2 Materials and methods

### 2.1 Bacterial strains, media, and cytokines

The strains sharing similar genetic backgrounds used in this study were *S. aureus* USA 300 (Community Acquired (CA); ST8; *spa*-type 008 *agr*-type I, MRSA, SCC-IV) and *S. aureus* ATCC 27543 (Livestock Associated (LA); ST8, *spa*-type 008; *agr*-type I, MSSA). Bacteria were inoculated in Trypticase Soy Broth (TSB) medium from BD-Bioxon, supplemented with oxacillin (50 ng/µL; USA300) and without antibiotic (ATCC 27543). To store *S. aureus* cells, TSB medium with 15% glycerol was used and kept at -70°C. The working *S. aureus* strains were inoculated in salt and mannitol medium (Bioxon) and stored at 4°C for short-term conservation. TSB was used in some cases, supplemented with 1% glucose, 1% sodium chloride, 1X PBS, and 1X TBS. Pro- and anti-inflammatory cytokines were added in a concentration range between 0.01–500 ng/mL diluted in PBS or TBS 1X, for biofilm formation experiments. Human recombinant cytokines (IL-1β, IL-10, and TNF-α) were obtained in lyophilized form from PeproTECH (Rocky Hill, NJ, USA). IL-1β, IL-10, and TNF-α were reconstituted in 5 mM Saline Phosphate Buffer or Saline Tris Buffer, pH 7.2 at 10, 10 and 50 ng/µL, respectively. Working aliquots were stored at -20°C.

### 2.2 Inoculum preparation

The bacteria were inoculated in 4 ml of TSB with or without oxacillin for 24 h at 37°C, and a dilution of the culture in the same medium was made to obtain 1.5 X 10^4^ CFU/mL. The absorbance (0.1) of each dilution was measured at 595 nm on a HALO DB-20 UV-VIS DOUBLE BEAM spectrophotometer.

### 2.3 Biofilm Formation Index and CFU/ml

To evaluate the effect of phosphates and cytokines on biofilm formation, bacteria were inoculated in 96-well polypropylene plates with 200 µl of bacterial culture in each well in TSB broth, supplemented with PBS (pH 7.0) at concentrations of 0.025X, 0.05X, 0.075X, and 0.1X for phosphate dilution effect experiments. 1% glucose and 1% NaCl were added as positive and negative controls, respectively, for biofilm formation. Pro- and anti-inflammatory cytokines were added at the beginning of each biofilm formation assay at the concentrations indicated in Materials and Methods; cells were incubated at 37°C for 24 h under static conditions. Supernatant was subsequently aspirated, and the wells were washed with 200 µl buffer saline (1X PBS pH 7.4). The bacteria from the biofilms and the supernatant were recovered for viable counts. For the evaluation of bacterial biofilms, they were recovered with 250 µL of 1X PBS, shaked with the tip of the micropipette and scraped from the walls. The viable count was carried out by drip plate sowing (24). For the colorimetric analysis of the biofilm, wells were stained as described by Christensen and colleagues (25). Briefly, the supernatant of the culture was removed and the plate was allowed to dry inverted at 37°C for 1 h. For staining, wells were fixed with 250 µL of methanol for 15 minutes. The methanol was removed, and it was allowed to dry for 10 minutes at 37°C. The biofilm was stained with 150 µL of a 0.1% (w/v) solution of crystal violet for 20 minutes at room temperature, rinsed with water until the excess dye was removed, and allowed to dry for 15 minutes. The dye on the attached (stained) bacteria was dissolved with 280 µL of absolute ethanol per well for 30 min. The plate was read in a plate spectrophotometer (Halo DB–20 UV-VIS Double Beam) at an absorbance of 595 nm. Biofilm formation index (BFI) was calculated as BFI = (B–C)/G, where B represents the crystal violet quantitation in OD units for each treatment, C is the crystal violet quantitation in control wells, and G is the absorbance of bacterial cell density in each well (26). Three independent experiments were performed with five replications each. Media of the five replicates were calculated and used in statistical analysis for an n = 3.

### 2.4 Analysis of biofilms with confocal laser scanning microscopy

The structure and distribution of viable cells in biofilms were analysed using the LIVE/DEAD^®^ BacLight™ staining system according to the manufacturer’s instructions. The LIVE/DEAD kit contains two nucleic acid markers, propidium iodide (PI) and SYTO9 (27). *S. aureus* USA 300 and *S. aureus* ATCC 27543 were grown in 4 mL of TSB with the corresponding antibiotics and incubated at 37°C for 24 hours. The pre-inoculum was diluted 1:200 in fresh broth to reach an optical density of 0.1 at a wavelength of 595 nm. 200 µL of each dilution was transferred to a 96-well plate. Treatments were added with IL-1β or IL-10 at concentrations of 1 ng/mL and 20 ng/mL, and TNF-α at 0.1 pg/mL and 1 ng/mL. The plate was incubated statically at 37 °C for 24 hours. The supernatant was discarded, and the biofilm formed was stained with SYTO 9 and IP. The images were obtained using the laser scanning microscope (CLSM) in a Flouview FV 1000 (Olympus) and were analysed with the Fiji ImageJ software. Measurements of biofilm thickness and live- and dead-cell zone areas were obtained,

### 2.5 Gene expression analysis of global regulators of transcription

Inoculum preparation and biofilm assays were performed as previously described. Total RNA from sessile bacterial cells was extracted as described by Atshan et al. (28). RNA was quantified in a NanoDrop 2000 spectrophotometer (Thermo Scientific) and evaluated for purity by reading the As_260nm_/As_280nm_ ratio. Integrity was verified in an electrophoresis in formaldehyde-containing 1% agarose. cDNA synthesis was performed with a First Strand cDNA Synthesis Kit (Thermo Scientific) as suggested by the fabricant. Gene expression of *agrA*, *RNAIII/hld*, *saeR*, *sarA*, and *sigB* was performed in a CFX96 Touch Real-Time PCR Detection System, Bio-Rad, using *gyrA* as a reference gene. Oligonucleotides for each gene are shown in **Table 1**. qPCR reactions were performed with qPCR, SsoAdvanced™ Universal SYBR^®^ Green Supermix kit (Bio-Rad) using 1 μg of total RNA, 800 nM of each oligonucleotide and 0.25 enzyme units. Thermocycler conditions were: initial denaturation at 94°C, 10 min followed by 30 cycles of 94°C, 15 sec; 63°C, 30 sec; 72°C, 1 min, with a final extension cycle at 72°C for 5 min (32, 33). Changes in RNA levels were determined by the ΔΔCt method (34). Data represents the mean of two independent experiments with two replicates each (n=4).

**Table 1:** Oligonucleotides used for qPCR assay.

Gene	Oligonucleotide	Sequence	Reference
*gyrA*	gyrAF gyrAR	5´ TGGCCCAAGACTTTAGTTATCGTTATCC ´3 5’ TGGGGAGGAATATTTGTAGCCATACCTAC 3’	(29)
*agrA*	agrAF agrAR	5’ AAGCATGACCCAGTTGGTAACA 3’ 5’ ATCCATCGCTGCAACTTTGTAGA 3’	(30)
*rnaIII*	rnaIIIF rnaIIIR	5’ GCACTGAGTCCAAGGAAACTAACTCT 3’ 5’ AGCCATCCCAACTTAATAACCATGT 3’	(30)
*saeR*	saeRF saeRR	5’ GTTGAACAACTGTCGTTTGATGA 3’ 5’ ACCACAATAACTCAAATTCCTTAATACG 3’	(31)
*sarA*	sarAF sarAR	5’ TTTTAACCATGGCAATTACAAAAAT 3’ 5’ TTTCTCTTTGTTTTCGCTGATGTAT 3’	(31)
*sigB*	sigBF sigBR	5’ TCAGCGGTTAGTTCATCGCTCACT 3’ 5’ GTCCTTTGAACGGAAGTTTGAAGCC 3’	(30)

### 2.6 Statistical analysis

Data were analysed with a one-way ANOVA, using a comparison with the Tukey test at a probability of p ≤ 0.05, for statistically significant differences. Statistical analysis was performed using GraphPad Prism 10 software (Graph Prism Software Inc, La Jolla, CA, USA).

## 3 Results

### 3.1 Standardisation of biofilm quantitation

Christensen (25) proposed a classification for the ability of biofilm formation based on the amount of biofilm adhered to plastic surfaces in tubes. Later, Stepanovic et al. (35) proposed modified methods for biofilm evaluation in plastic plates and proposed a classification on the ability to form biofilm as non-adherent (As595nm = 0), weak (As595nm ≤ 0.12), moderate (0.12 ≤ As595nm ≤ 0.24) or strong (As570nm > 0.24). According to our data, *S. aureus* USA 300 is a strong biofilm former (As595nm = 0.35) and ATCC 27543 is a medium-to-strong biofilm former (As595nm = 0.25). This is also in accordance with Lade et al. (36), who reports that methicillin-resistant *S. aureus* bearing Staphyococcal Chromosomal Cassette *mec* IV (MRSA-SSC*mec*IV strains; as USA 300) are better biofilm formers than Methicillin-sensitive *S. aureus* strains (MSSA; as ATCC 27543).

**Figure 1:**
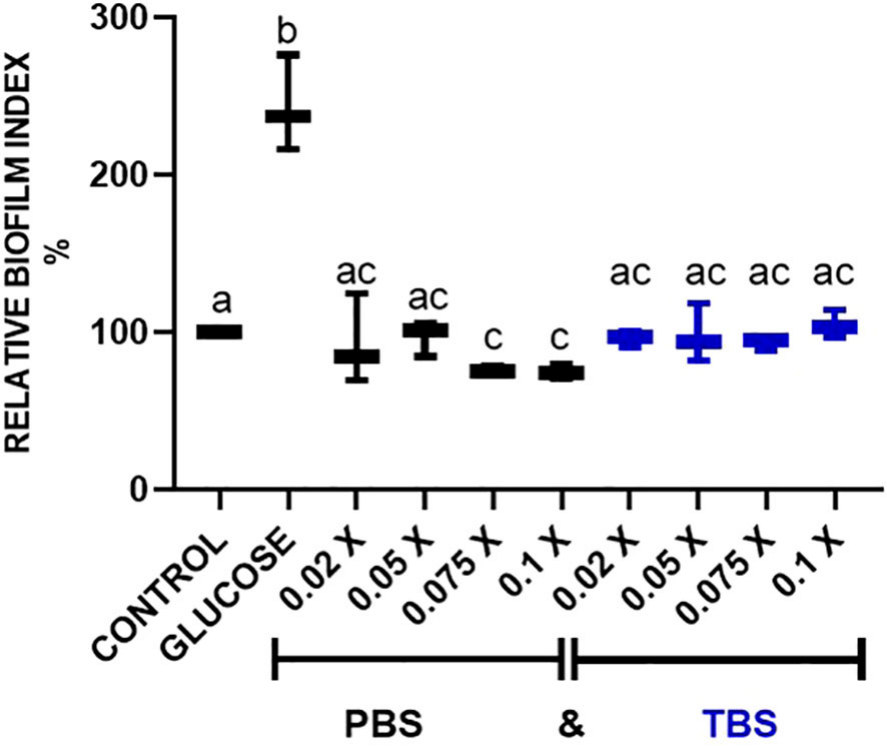
Effect of PBS dilution of biofilm formation. *S. aureus* USA 300, cells treated with different dilutions (0.025 X, 0.05 X, 0.075 X, 0.1 X) of phosphate-buffered saline (1 X PBS) or TRIS-buffered saline solution (1 X TBS). Values with different letters indicate statistically significant differences (*p* < 0.05) among treatments.

It has been reported that biofilms are affected by different stress conditions, temperature, pH, nutrients, among others. In preliminary assays, we observed that PBS dilution, when added to the medium in biofilm-forming experiments, altered biofilm formation. So, we decided to test if there was an effect of PBS dilution in biofilm formation and compare it with the use of TBS. In **Figure 1** biofilm formation index for the *S. aureus* USA 300 was evaluated with different dilutions of PBS (0.1X, 0.075X, 0.05X, 0.025X), which correspond to 230.4 µg/mL, 173.3 µg/mL, 115.2 µg/mL, 57.6 µg/mL of Na_2_HPO_4_ and KH_2_HPO_4,_ respectively, compared to a phosphate-free tris buffer saline (TBS 1 X). The results demonstrate that there is a dose-dependent effect of the PBS dilution used in biofilm formation. In **Figure 1A** decrease in biofilm formation was observed when increasing concentrations of phosphates. *S. aureus* USA 300 showed a 30% inhibition of biofilm formation index, while *S. aureus* ATCC 27543 showed a 50% inhibition (**Supplementary Figure S1**).

### 3.2 Cytokines decrease cell viability in sessile and planktonic cells in biofilms of *S. aureus.*

**Figure 2:**
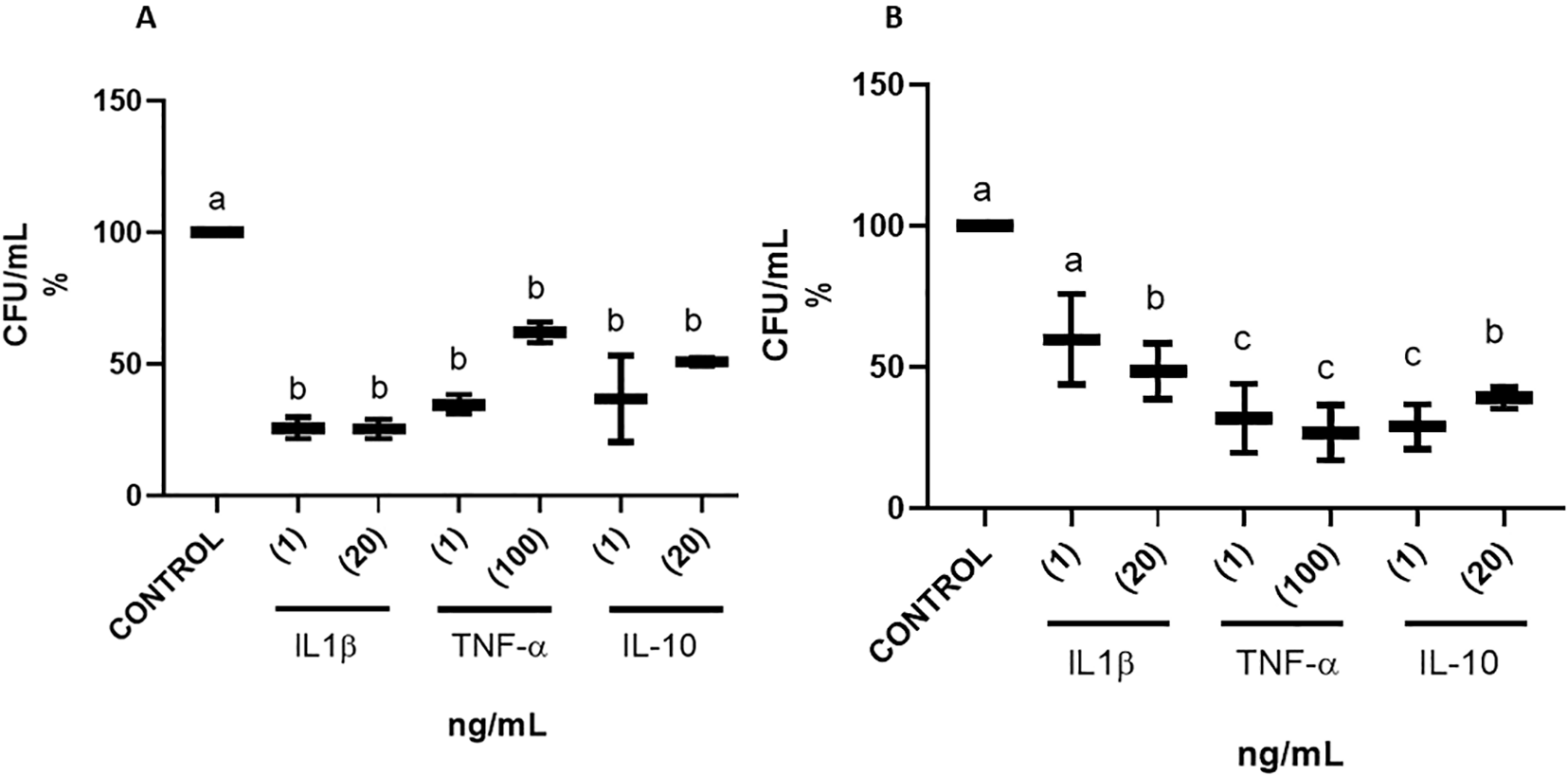
Cell viability in biofilms of *S. aureus* in response to cytokines. *S. aureus* USA 300. **(A)** CFU/ml of the sessile phase of the biofilm, and **(B)** CFU/ml of the planktonic phase. Different letters indicate significant differences. The number in parentheses in the graph indicates the concentration of the cytokines in ng/ml. Values with different letters indicate statistically significant differences (*p* < 0.05) among treatments.

To analyse the effect of cytokines on *S. aureus* strains during biofilm formation, we evaluated the viability of both sessile (attached) and planktonic (suspended) bacterial cells through viable counts, by using two concentrations of proinflammatory (IL-1β; 1.0 and 20 ng/mL and TNF-α; 1.0 and 100 ng/mL) and anti-inflammatory (IL-10; 1.0 and 20 ng/mL) cytokines. For *S. aureus* USA 300, TNFα, IL1-β and IL-10 decreased sessile cells viability to a minimum of 31%, 29% and 52%, respectively, as compared to the control. Cytokines also decreased planktonic cell viability to 42%, 34% 41%, respectively, with respect to the control. (**Figures 2A, B**). For *S. aureus* ATCC 27543, TNFα, IL1-β and IL-10 decreased sessile cells’ viability to a minimum of 60%, 42.5% and 50%, respectively, in comparison to the control. Cytokines also decreased planktonic cells’ viability to 43%, 31%, 30%, respectively, as compared to the control. 1% Glucose and 1% NaCl were used throughout the experiments as positive and negative controls of biofilm formation, respectively (36, 37); **Supplementary Figures S2A, B**). These results suggest that pro- and anti-inflammatory cytokines reduce the viability of both sessile and planktonic cells in the biofilms.

### 3.3 Cytokines decrease relative biofilm index formation in *S. aureus*

**Figure 3:**
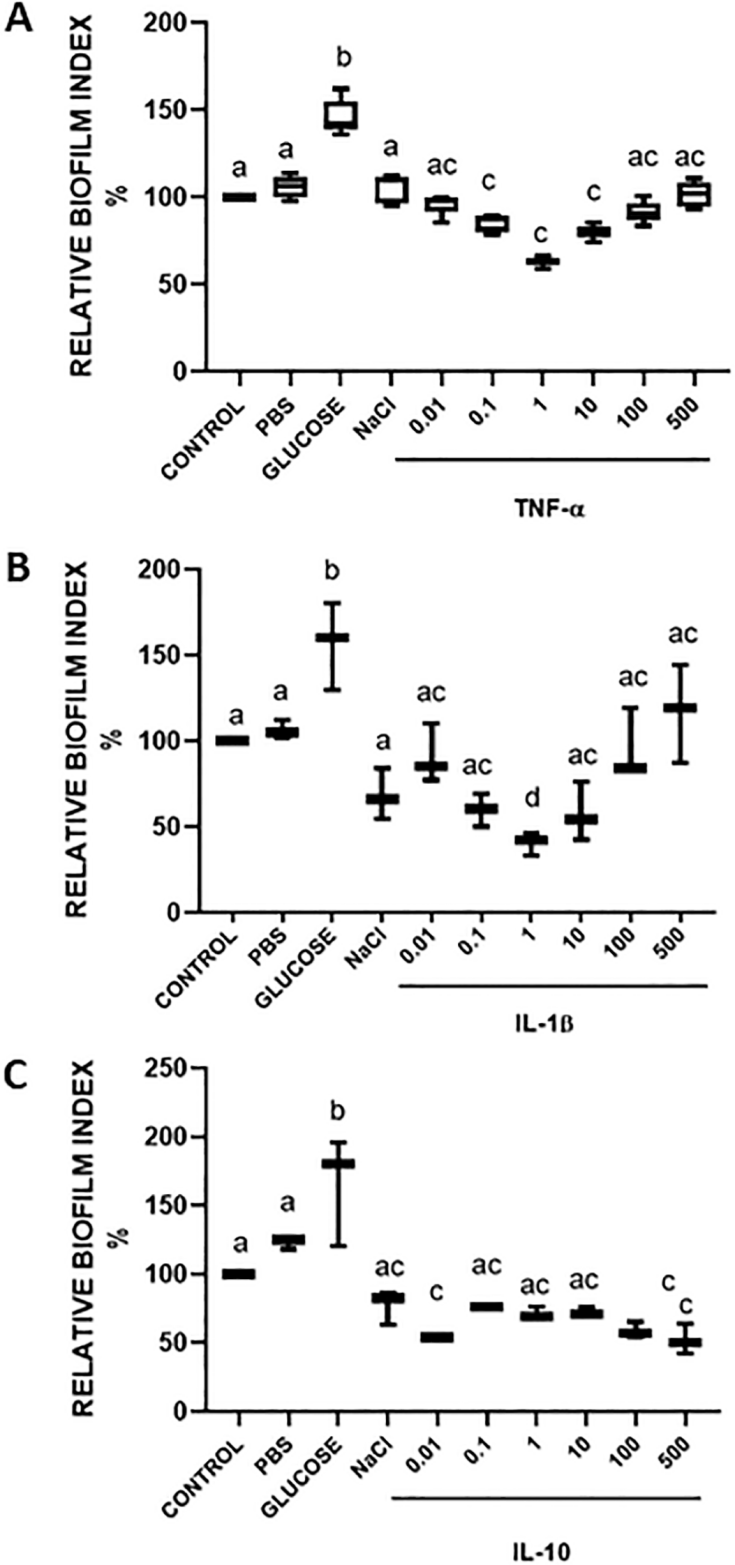
Dose–response effect of cytokines on *S. aureus* biofilm formation. *S. aureus* USA 300 incubated with TNFα **(A)**, IL-1β **(B)** or IL-10 **(C)**. Values with different letters indicate statistically significant differences (*p* < 0.05) among treatments.

It has been shown that cytokines concentrations may range from pg/ml in circulating blood to hundreds of ng/ml in infection or inflammation sites, such as cerebral-spinal fluid in meningitis and septic shock (38), bronchial lavage in ventilator-associated or community-acquired pneumonia (39, 40), and in pus samples from hidradenitis suppurativa (41). So, to test the responsiveness of *S. aureus* to cytokines, dose-response assays were performed using concentrations from 10 pg/ml to 500 ng/ml (**Figure 3**; **Supplementary Figures S3**). Glucose has been used as a positive inducer of biofilm formation (36). NaCl has been reported to not induce biofilm formation in Methicillin-Resistant *Staphylococcus aureus* bearing the Staphylococcal Chromosomal Cassette *mecI*V (MRSA-SCC*mec*IV) strains, a genotype similar to *S. aureus* USA 300 strain, so we used NaCl as a negative control (36). All the cytokines tested promoted a decrease in biofilm formation index, independently of their nature as pro-inflammatory or anti-inflammatory cytokines. For TNFα effect, BFI showed a peak of inhibition at 1 ng/ml in *S. aureus* USA 300 (**Figure 3A**), while for *S.aureus* ATCC 27543, a peak of inhibition was observed at 0.1 to 10 ng/ml (**Supplementary Figure S3A**). Both responses were not linear, showing specific concentrations for maximum responses in each strain. IL-1β caused maximum inhibition at 10 ng/ml and 1 ng/ml in *S. aureus* USA 300 (**Figure 3B**) and ATCC 27543 (**Supplementary Figure S3B**), respectively. It is interesting to note that none of these responses were linear, suggesting the presence of receptors or target proteins that behave like a protein-ligand interaction kinetics. It is also interesting that the response of *S. aureus* ATCC 27543 was bimodal, while USA 300 did not show this response. These suggest that different mechanisms of perception of IL-1β may be acting for each strain. The effect of IL-10 on both *S. aureus* strains was interestingly more differential between them than for the other analysed cytokines. For *S. aureus* USA 300 (**Figure 3C**), a bimodal behaviour of inhibition was observed, with maximum effects at 0.01 and 500 ng/mL. For *S. aureus* ATCC 27543 (**Supplementary Figure S3C**), a peak of inhibition was observed between 0.1 to 1.0 ng/mL of IL-10, suggesting again subtle differences in the possible mechanisms for IL-10 perception between the strains.

### 3.4 Cytokines differentially alter the structure of *S. aureus* biofilms

**Figure 4:**
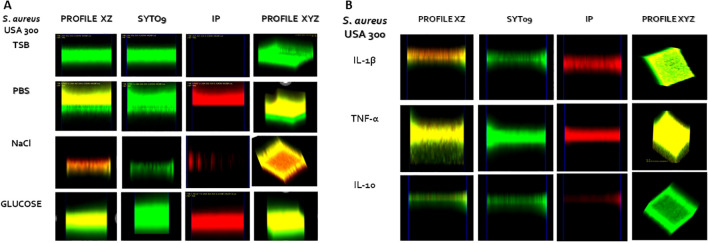
CSLM of *S. aureus* USA 300 biofilms under the effect of immunomodulatory signals. Panel **(A)** experimental controls; panel **(B)** cytokine treatments. The treatments used were TSB alone or supplemented with 0.01 X PBS, 1% glucose, 1% NaCl, IL-1β, TNF-α and IL-10. XZ profiles showing, from left to right, merged images, SYTO9 channels and PI channels. XZY profiles show only the merged images. Images are representative of at least two replicates.

Confocal laser scanning microscopy (CLSM) is one of the most widely used tools to study biofilm structure. It has the advantage of allowing the capture piles of images in different optical planes to be integrated into 3D images and to capture images of living and dead cells. The CLSM images of the biofilms formed by *S. aureus* USA 300 or *S. aureus* ATCC 27543, under the effect of cytokines, are depicted in **Figure 4** and **Supplementary Figure S4**, respectively.

IL-1β, TNFα, and IL-10 caused a decrease in cell viability and biofilm formation as we described above. CLSM micrographs are in agreement with those results, showing a general reduction in biofilm thickness when treated with the cytokines and an increase in the proportion of dead cells in the biofilm structure. However, several interesting differences between *S. aureus* strains and cytokines effects were observed. In the case of *S. aureus* USA 300 (**Figure 4A**) a zone of dead cells into the biofilm is observed both, in the presence of PBS and NaCl as compared to the strain grown on TBS medium or stimulated with glucose, in which there were not observed zones of dead cells. For PBS, the zone of dead cells was in the upper region of the biofilm, while for NaCl, it was observed in the middle region of the biofilm. The same was observed for *S. aureus* ATCC 27543 (**Supplementary Figure S4A**) except that the treatment with PBS did not show a zone of dead cells.

**Figure 5:**
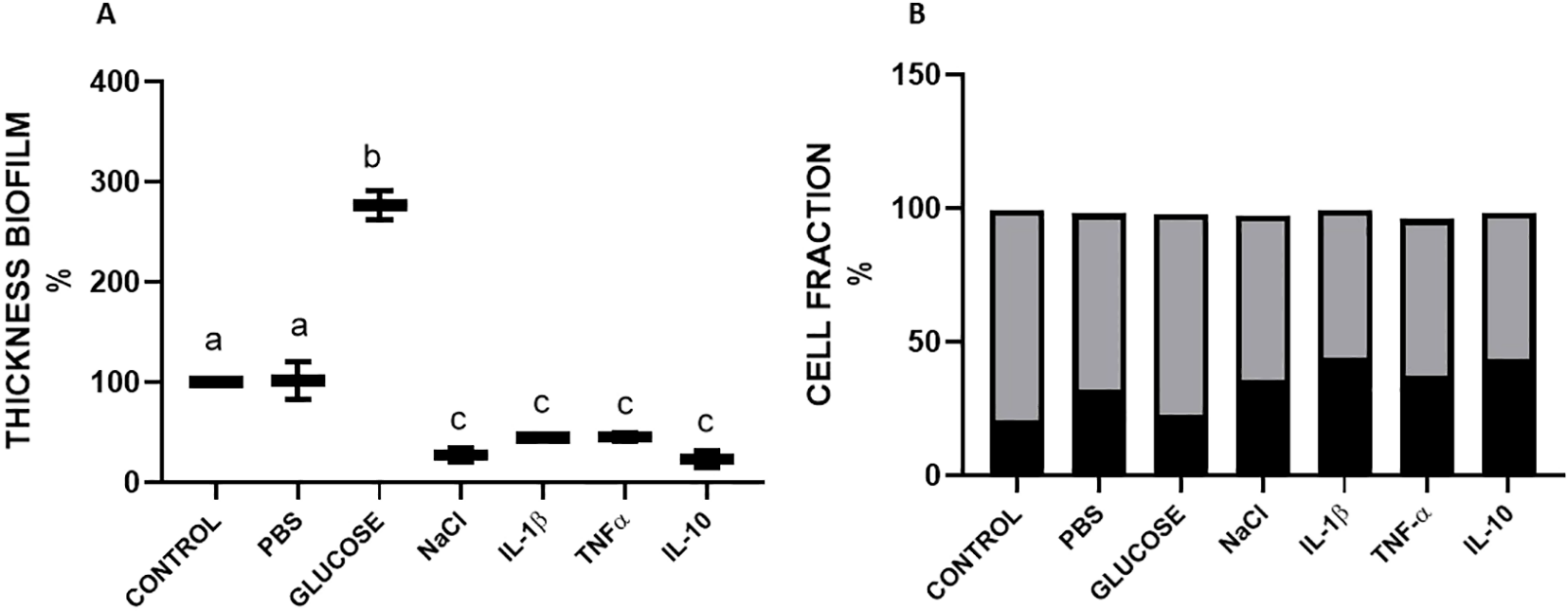
Thickness of *S. aureus* biofilms. *S. aureus* USA 300, was stained with fluorescence and captured with the laser scanning confocal microscope and measured with the ImageJ program. **(A)**, Thickness of the biofilm as a percentage of the control. **(B)**, fractions of live (gray bars) and dead (black bars) cells of *S. aureus* USA 300. Plotted data are representative image areas from two experiments. The proinflammatory cytokines IL-1β, TNF-α were used at 10 and 1 ng/ml respectively; IL-10 was used 10 ng/ml. Values with different letters indicate statistically significant differences (*p* < 0.05) among treatments.

To get a better picture of the behaviour of biofilm structure under the different stimuli, we measured the biofilm thickness using an XZ view cut of the central region of the biofilm and measured the proportion of biofilm thickness that contained viable or dead cells (**Figure 5**; **Supplementary Figure S5**). For *S. aureus* USA 300, 0.01 X PBS treatment did not show significant differences in thickness, while increases and decreases in thickness induced by 1% glucose or 1% NaCl were statistically significant at *p* < 0.05 (**Figure 5**). For *S. aureus* ATCC 27543 there were no statistically significant differences at *p* < 0.05 for 0.01X PBS or 1% NaCl, but the increase in thickness by 1% glucose was statistically significant (**Supplementary Figure S5**). Regarding the effects of cytokines, all three cytokines previously showed an inhibitory effect on biofilm formation and on cell viability. This was in accordance with CLSM observations in which all cytokine treatments showed an increase in the proportion of dead cells (**Figure 5**; **Supplementary Figure S5**). However, for *S. aureus* USA 300, all IL-1β, TNFα and IL-10 showed statistically significant reductions of thickness as compared to the TBS control. (**Figure 5A**). For ATCC 27543, only with the treatment with TNFα, the reduction in biofilm thickness (**Supplementary Figure S5A**) resulted in a statistically significant difference (*p* < 0.05). To assess the effect of the treatments on cell viability visualised by CLSM, we calculated the proportion of PI-stained thickness with respect to the total thickness of the biofilm (**Figure 5B**; **Supplementary Figure S5B**). For both strains, controls showed similar proportions (less than 25%) of dead cells. The behaviour of the positive and negative controls, as well as of the treatments with cytokines, was similar among the strains. In both cases, the greater proportion of dead cells was observed for the IL-1β treatment. Another interesting observation is the fact that *S. aureus* ATCC 27543 biofilms showed greater continuity, than *S. aureus* USA 300, in which biofilm staining showed discontinuities. These discontinuities in staining may be related to the presence of channels into the biofilm structures (**Figure 4**; **Supplementary Figure S4**). The effect on discontinuity of staining in the biofilm, particularly for TNFα, was more evident for the three cytokines in the *S. aureus* USA 300 strain than in ATCC 27543. Interestingly, this observation correlates with the greater effect on biofilm thickness in *S aureus* USA 300.

### 3.5 Cytokines differentially alter gene expression of global regulators of transcription in *S. aureus* biofilms

**Figure 6:**
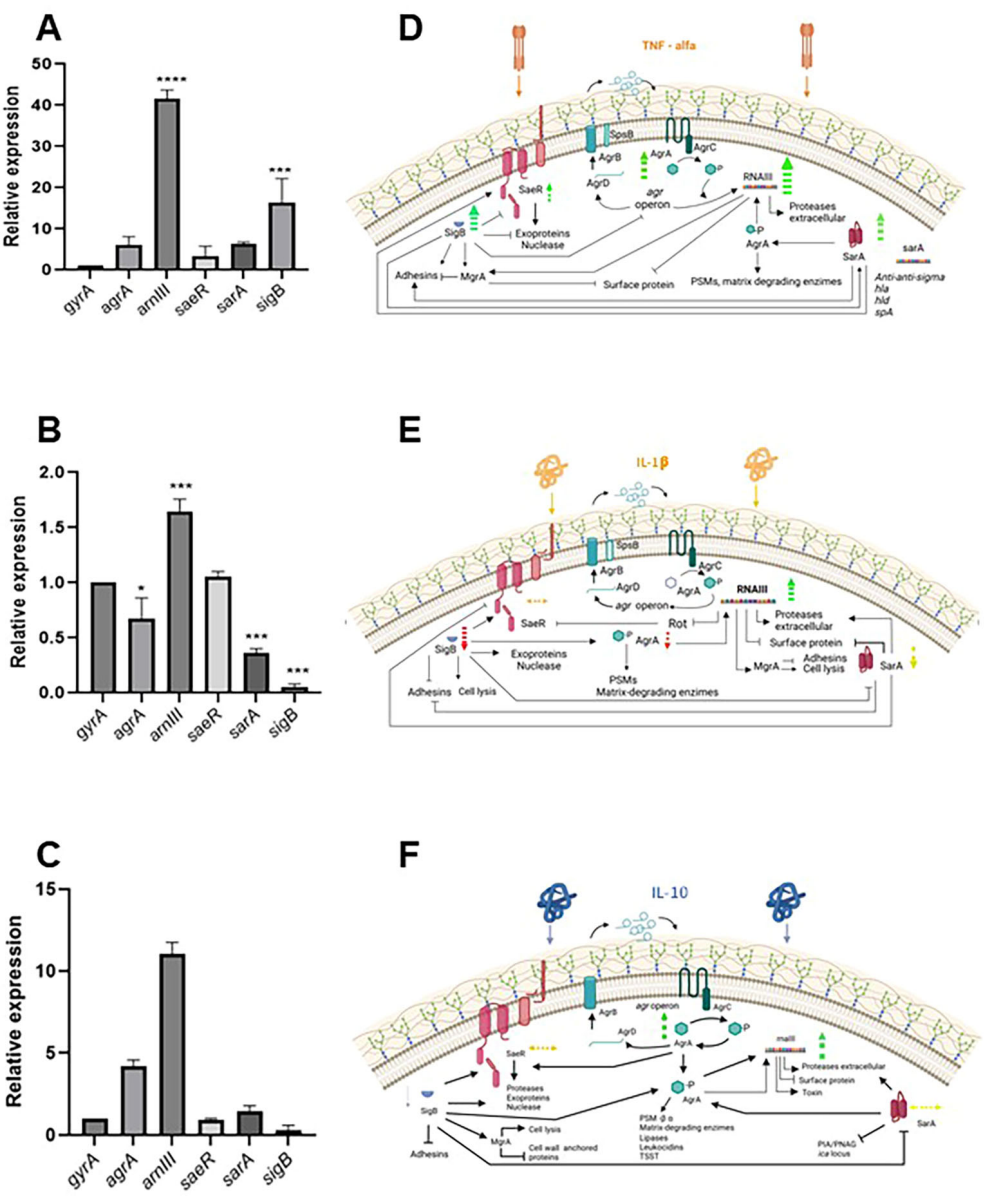
Effect of cytokines on the expression of global regulators of biofilm formation. Relative gene expression levels of global regulators of biofilm formation. Stimulated with TNFα **(A)**, IL-1β **(B)** or IL-10 **(C)**. Statistically significant differences are as follows; * *p* < 0.05; *** *p* < 0.0005; **** *p* < 0.0001. Models suggesting possible relationships between cytokine effects on global regulators of biofilm formation: TNFα **(D)**, IL-1β **(E)**, or IL-10 **(F)**. Green or red discontinuous arrows show increases or decreases in relative gene expression on the indicated gene, respectively; yellow arrows with two arrowpoints represent no significant changes in gene expression. Solid lines represent regulatory interactions, either positive (arrowpoints) or negative (endline).

Results of gene expression levels during cytokine treatment in biofilm formation in the *S*. *aureus* USA 300 strain are shown in **Figure 6**. We selected only the *S. aureus* USA 300 strain for gene expression assay because cytokines are of human origin, the strain is a human pathogen and previous results on bacterial growth inhibition and biofilm formation and structure were clearer and more consistent with this strain. When TNFα was present during biofilm formation, expression levels of *rnaIII* and *sigB* were significantly increased (41.6- and 16.4-fold change, respectively; **Figure 6A**), while *agrA*, *saeR* and *sarA* showed non-statistically significant increases. When IL-1β was present during biofilm formation, *rnaIII* showed a statistically significant increase in expression levels (1.6-fold change), while *agrA*, *sarA* and *sigB* decreased their expression levels significantly (0.7-, 0.4- and 0.05-fold change, respectively); *saeR* didn’t show a significant change in expression level (**Figure 6C**). The effect of IL-10 on gene expression during biofilm formation showed an increase in *rnaIII* and *agrA* expression levels (11.0- and 4.2-fold change, respectively) and a decrease in *sigB* expression level (0.3-fold change; **Figure 6E**). The specific changes in gene expression levels for each cytokine suggested that there may be a different sensing mechanism and possibly signal transduction pathways for each cytokine.

## 4 Discussion

Biofilm is the prime barrier of pathogens to deal with environmental stress, immune response, and antimicrobial molecules, among others. It is also one of the early pathogenic features shown by bacterial pathogens. It helps the bacteria to attach to biological or inert surfaces as part of the initiation of an infection process. As an inflammatory-inducing opportunistic pathogen, *S. aureus* stimulates, via its surface molecules, the onset of innate immune response, turning on cytokine signalling to recruit cellular defences. Therefore, a thorough understanding of the initial interaction between S. aureus and the host’s immune system is essential for reducing and controlling infections at their earliest stage. The present study focused on the analysis of the effect of signals from the host, the early-expressing cytokines IL-1β, TNFα (pro-inflammatory) and IL-10 (anti-inflammatory), on the formation of one of the early-expressing virulence traits of *S. aureus*, its biofilm. In our assays, when establishing conditions to add cytokines to the biofilm, we noticed that increasing the concentration of phosphates led to a reduction in biofilm formation. A similar observation was reported by Ghosh and colleagues in *Burkholderia tropica* P4 and *B. unamae* P9 strains, about the effect of soluble and insoluble phosphates (42). Since the addition of PBS, the phosphate buffer saline solution used to dilute the molecular stimuli added to the biofilm, did cause a reduction (**Figure 2**) we decided to try TBS (Tris buffer-saline) solution to dilute the molecular stimuli. No reduction of the ability to form biofilms was shown for TBS in none of the *S. aureus* strains. So, in our experiments, PBS was diluted at a final concentration of 0.01X or lower, or TBS was used as diluent.

The addition of cytokines IL-1β, TNFα, and IL-10 to *S. aureus* strains resulted in a decrease of cell viability for both strains in both sessile and planktonic bacterial cells. For *S. aureus* ATCC 27543, higher concentrations of IL-1β resulted in a higher inhibition. This was not the case for TNFα and IL-10, for which both concentrations (1 and 100 mg/mL and 1 and 20 ng/mL, respectively) did not show differences in the inhibition of cell viability, neither in sessile nor planktonic cells. This may suggest that there is a specific signal-receptor system for IL-1β. Although an inhibition of sessile and planktonic growth was observed for all cytokines, we do not perform tests to evaluate possible bacteriostatic or bactericidal effects, because biofilm formation was evaluated 24 hours after cytokine exposure. Even if alteration in biofilm formation and reduction in cell viability are the long-term consequence of cytokine exposure, it would be interesting to test if cytokines themselves present bacteriostatic or bactericidal activities. Several reports suggest that cytokines may affect cell viability and growth. Meduri and colleagues reported that *S. aureus* isolates from bronchoalveolar lavage fluid or peripheral blood enhanced growth in CDM medium with IL-1β, IL-6 and TNFα when applied at concentrations of 1 and 10 ng (43). In *S. aureus* an increase in growth has been reported in the presence of recombinant IL 1-β in a concentration-dependent manner, and this is reversed with a 25-fold excess of an IL-1 receptor agonist (44). Several reports have suggested that in either Gram-positive or Gram-negative bacteria may exist cytokine specific receptors (44–46).

The effects of cytokines on biofilm formation in our work revealed that there are specific response peaks at different concentrations for each cytokine. All concentration-dependent responses present sigmoidal behaviour, with a maximum response concentration (all three cytokines in *S aureus* USA 300 and IL1-β in *S. aureus* ATCC 27543) or a range of concentrations of maximal response (TNFα and IL-10 in *S. aureus* USA 300). The presence of peaks or a delimited range of concentrations for maximum response to cytokines is in accordance with the possible presence of specific receptors to each cytokine, as it has been previously suggested (43–45, 47). To the best of our knowledge, not a single receptor to cytokines has been described in any bacteria. However, it has been reported that cytokine receptors in human and bovine hosts share structural similarity and are responsive to cytokines. Bovine TNF receptor I (TNF-RI) shares 67% identity of aminoacid sequence with human receptor. It also shares conserved cysteine domains and glycosylation residues that are essential for its function in humans (48). Bovine IL-1 receptor II (IL-1rII) also shares high identity (71%) and similarity (86%) levels with human receptor, as well as conserved cysteine and proline residues, glycosylation sites and a putative transmembrane motif also present in mouse and rat IL-1rII (49). To our knowledge, there are not reports on molecular characterization of bovine IL-10 receptors. There are also several reports on conservation and evolutionary relatedness of cytokine receptors in mammals (50, 51). There are also several publications in which human cytokines have been used in bovine cells (23). There are differences in our results with those from other groups reporting growth enhancement (43–45, 47), or promotion of biofilm formation. McLaughlin and Hoogewerf demonstrated that IL-1β enhances the growth of sessile cells but not on planktonic ones in *S. aureus*, and this stimulation correlated with the ability to bind ^125^I-IL-1β (52). These suggest once again that IL-1β may have a specific receptor in bacterial cells. In a mouse mastitis model, Gogoi-Tiwari and colleagues showed that the intensity of inflammation in the mammary gland, measured as the levels of cytokines, does not correlate with the ability of non-typeable *S. aureus* clinical isolates to form biofilm (53). It has been shown that human monocytes (U937 cells) when treated with high concentrations of LPS, increase the levels of proinflammatory cytokines (IL-1β, TNF-α and IL-6), favouring the development of *S. aureus, P. aeruginosa, and Acetobacter* spp., and when using methylprednisolone, a significant reduction in bacterial replication was observed in a concentration-dependent manner (43, 54). In another study carried out on bovine epithelial cells pretreated with IL-1β and TNF-α, it was determined that the level of internalization of *S. aureus* increase in a dose-dependent manner: increasing the cytokine concentrations also increases the degree of internalization, in addition to verifying the participation of the nuclear transcriptional factor kappa beta (NF-Kβ) in the promotion of internalization (23). Another report (55) shows an increase in bacterial biofilm in *S. aureus* Xen29 when stimulated with IL-1β, which is in contrast with our result of biofilm inhibition by IL-1β. McLaughlin et al. (52) demonstrated that *S. aureus* strains ATCC 12600 and ATCC 25923 increased growth when stimulated with IL-1β. These discrepancies may be related to *S. aureus* genetic background, as it has been suggested for NaCl response among *S. aureus* isolates (36). *S. aureus* Xen29 is a transgenic strain modified for bioluminescence detection which has not been previously genotyped. ATCC 12600 is an MRSA isolate that has not been genotyped, and ATCC 25923 is an MSSA strain genotyped as ST243, which is a singleton (as determined on PubMLST web page), an ST not grouped with other known STs, so, genetically different from our strains. Except for ATCC 25923, we have no evidence that Xen29 or ATCC 12600 may be or may be not related genotypically with the strains we used. So, we believe that growth inhibition in *S. aureus* USA 300 and ATCC 27543 by IL-1βmay be due to different genotypic backgrounds. Overall, these studies, when contrasted with our results, suggest that the effect of cytokines on the ability to form biofilms on *S. aureus* strains depends on the environmental context, the genotype and physiological state of the bacteria.

Meduri and colleagues (43) demonstrated that the degree of monocyte activation, induced by increasing concentrations of the proinflammatory cytokines TNF-α, IL-1β, and IL-6 or lipopolysaccharide (LPS), modulated the intracellular growth of *S. aureus*, *P. aeruginosa*, and *Acinetobacter* spp. The intracellular growth of bacteria in monocytes was decreased by low concentration of cytokines (10 to 250 pg). At higher concentrations of cytokines or LPS (1 to 10 µg), monocytes intracellular growth was increased. Another study reports that when measuring the growth of *S. aureus* in a simple tissue culture medium with minimal nutrients (RPMI), a dose-dependent growth was observed in the presence of IL-1β (10 pg - 100ng), but not when using TNF- α, nor IL-6. These observations suggest that bacteria could be detecting cytokines through a receptor-mediated signal transduction mechanism that require similar biochemical processes than those described in eukaryotic cells (43). There is also the possibility that break-down products of cytokines may act as biologically active molecules that directly or indirectly may control transcription and translation of specific genes (43).

Engelsöy and colleagues tested proinflammatory cytokines (TNF-α, IL-1β, IL-6, IL-8 and IFN-γ); they observed a decrease in the survival of *Caenorhabditis elegans* by altering the virulence of *E. coli* (56). In mice implanted with osmotic pumps, treated with different cytokines and colonised with *S. aureus* Xen29 before implantation in mice, it was reported that IL-1β at a concentration of 83 µg/mL favours the biofilm formation of *S. aureus*, as well as the massive influx of neutrophils adjacent to the cells with biofilm-coated pumps *in vivo*. In this same study, TNF-α and IL-10 were used, but they did not affect biofilm formation (55). For *E. coli*, but not for *P. aeruginosa*, *in vitro* proliferation was increased by recombinant mouse TNFα and the effect was blocked by anti-TNFα antibodies (57). TNFα alters microbial composition and microbial activities, attenuating colorectal carcinogenesis in mice, suggesting that the microbiota is susceptible to signals emitted by the host (58). Likewise, host cells can sense the presence of microorganisms; this communication was exemplified in the study by Bhardwaj and colleagues (59), who stimulated human blood cells with polymicrobial biofilms.The anti-inflammatory cytokine (IL-10) decreased bacterial growth in both strains of *S. aureus*, as well as the formation of biofilms, also generating a decrease in the thickness of the biofilm structure and probably an increase in the formation of channels in the biofilms developed *in vitro*. There are only reports of the modulatory action of IL-10 in infections caused by *S. pneumoniae, P. aeruginosa, M. tuberculosis*, and *F. tularensis*, which is necessary for the host survival during infection; IL-10 promotes downregulation of inflammation to prevent excessive cell damage without interfering with bacterial clearance. However, the suppression of the immune response by high levels of IL-10 may facilitate bacterial propagation, thus increasing the severity of the disease. Since *S. pneumoniae* and *P. aeruginosa* are extracellular bacteria, they require several virulence factors for the neutralization of the host’s immune response. IL-10 also alters the bactericidal ability of the host, promoting growth, bacterial dissemination, and survival of the intracellular bacteria *B. pertussis, B. abortus*, *K. pneumoniae, L. monocytogenes*, and *S. enterica*. The data obtained indicate that the absence of IL-10 leads to a more efficient bacterial elimination, a lower rate of dissemination, and a greater host survival (60). B cells that produce IL-10 upon *S. aureus* activation via TLR2 play a crucial protective role against systemic dissemination, thereby reducing the associated morbidity and mortality. On the other hand, during local infection, IL-10 facilitates the survival of the bacteria (61). Penaloza and colleagues reported a differential production of IL-10 induced by intracellular multidrug-resistant (MDR) bacteria, altering host survival and bacterial clearance by increasing IL-10 production (62). During infections with extracellular or highly inflammatory bacteria, IL-10 production is needed for host survival (62). Given that IL-10 can exert both beneficial and detrimental effects on the host, further investigation is warranted into its potential use as a mechanism for bacterial control or elimination. It has been reported that *S. aureus* biofilms confer protection against antimicrobial therapy and immune elimination such as neutrophil extracellular traps (NET), secretion of haemolysin, nucleases and phenol-soluble modulins are important during the evasion of the immune system in the sessile state in the biofilms and could change the immune response to an anti-inflammatory state (12), so it is necessary to deepen in the study of the pathogen-host interaction. Recently, van Roy and Kielian (63) reviewed the relations between cytokines and biofilm formation on several infectious diseases, as prosthetic joint infection (PJI), craniotomy infection (CRAN), cerebral-spinal fluid infection (CSF) and catheter-associated infection (CATH). They describe that TNFα reduces bacterial numbers in PJI, but does not affect bacterial numbers in CRAN. IL-1β reduces bacterial numbers in PJI, CATH, and CRAN. IL-10 enhanced bacterial burden in PJI and CRAN, but does not affect bacterial burden in CSF. In view of these findings, it is suggested that the response of bacterial biofilms to cytokines is also subject to modulation by the specific microenvironmental (tissular) context.

Gene expression levels of global regulators of expression were also differentially affected by either TNFα, IL-1β or IL-10 in this work. Although all cytokines successfully induced *rnaIII*, TNF- α exhibited a markedly stronger induction, with expression levels exceeding those of IL-1β and IL-10 by more than 10-fold. A significant rise in *agrA*, and *sigB* relative expression levels in the presence of TNFα is contrasting with the decrease in expression levels caused by the presence of IL-1β or the lack of response of *sigB* in the presence of IL-10. These differential patterns of gene expression led us to propose models for the mechanism of action of each cytokine.

### 4.1 Effect of cytokines on *S. aureus* biofilms

**Figure 6D** depicts a proposal of how the observed changes in biofilm formation and relative gene expression integrate to explain the effect of TNFα. *rnaIII* and *sigB* were the genes with a higher increase in relative expression levels, and slight but not significant decreases for *agrA, sarA* and *saeR*. RNAIII functions as an antisense RNA, simultaneously repressing the expression of surface proteins (e.g., Spa, Sbl, and Coa) and inducing extracellular proteases and nucleases that degrade the biofilm’s extracellular matrix. These observations are in accordance with the presence of discontinuous fluorescence signals in biofilm structure as observed with CLSM. These discontinuities in biofilm structure may be associated with the presence of channels of the biofilm, which may be generated by extracellular hydrolytic enzymes. The global regulator MgrA, known to limit biofilm formation, is also induced by RNAIII. This RNAIII-mediated MgrA induction may consequently promote the increase in dead cells observed in Confocal Laser Scanning Microscopy (CLSM) images. *spa* mRNA, an early-expressing gene in biofilm formation, may be degraded because of ARNIII antisense action (1, 64–66). *sigB* relative gene expression was also increased by TNFα. SigB is known as an alternative sigma factor that is present in response to different forms of environmental stress. It has a dual role in biofilm formation, both in the initial attachment of bacterial cells to the surface and in promoting biofilm disassembly. SigB may act to decrease SaeR levels, which also induces MgrA causing adhesin repression. SigB may also stimulate AgrA via SarA, increasing PSMs and gene expression of the matrix-degrading enzymes. Increased levels of SigB and RNAIII may also promote biofilm degradation via AgrA and SaeR induction (1, 64, 65, 67, 68). *saeR*, which shows a slight induction in the presence of TNFα may be induced by *sarA* induction. The observed biofilm structure in the presence of TNF-α is consistent with the established role of the SaeRS system as a major regulator of biofilm maturation and dispersal.

IL-1β effect on *S. aureus* biofilm formation and relative gene expression levels is depicted in **Figure 6E**. Changes in relative levels of gene expression were not as intense for IL-1β as for TNFα. IL-1β slightly but significantly induced *rnaIII*, while repressed *agrA* and *sarA*, with a higher repression for *sigB*. The alternative stress-response Sigma factor, SigB, plays a dual role in promoting biofilm formation: it enhances bacterial adhesion and the production of Polysaccharide Intercellular Adhesin (PIA), and concurrently negatively regulates the proteases and nucleases necessary for late-stage biofilm dispersal.The reduction of *sigB* expression levels is consistent with our observations of a decrease in biofilm formation index, a reduction in biofilm thickness, an increase in the proportion of dead cells and the presence of discontinuity of fluorescence signal in CLSM analysis. *agrA* and *sarA* were also repressed in the presence of IL-1β. Both regulate the early stages of biofilm establishment; this is also consistent with the observed biofilm phenotype.

IL-10 increased expression levels of both *arnIII* and *agrA*. As discussed before, RNAIII has an important role in biofilm dissemination.

## 5 Conclusions

Microbial endocrinology studies on the role of cytokines in biofilm formation and structure may shed light on their possible role *in vivo.* This work, as compared to those reported in literature, seems to differ in the direction of the responses to cytokines, whether they increase or decrease cell viability or biofilm formation capacity. We selected two *S. aureus* strains from different pathologies, bovine mastitis and skin and soft tissue infections in humans, that share a common genetic background. There is a great similarity in responses to the cytokines TNFα, IL-1β, and IL-10 in both of the strains we selected; also, minor differences were observed in the kinetics of responses and in the fine structure of the biofilm between our strains. This is in accordance with our previous comments suggesting that the response to cytokines may be dependent both on the environmental context and on the background of the bacteria. Interesting differences in response to cytokines were observed at the structural level of biofilms, as observed in CLSM and in the pattern and intensity of relative gene expression of global regulators of virulence. These findings, combined with the previous observation of cytokine-specific sigmoidal response patterns and distinct peaks at varying concentrations, lead to the hypothesis that the observed S. aureus response is highly specific. This may be due to the engagement of different bacterial receptors or the activation of distinct signaling pathways by each cytokine. The overall landscape of biofilm formation in *S. aureus* in response to cytokines present at the onset of inflammation suggests that *S. aureus* can detect the inflammatory response through increases in cytokine levels and change its gene expression patterns to promote biofilm disaggregation, hence promoting dispersion of bacterial cells to other sites in the organism.

In the near future, new strategies to control *S. aureus* infections must be developed individually for each cytokine. More research to explain the differences in responses of *S. aureus* strains to cytokines must be conducted. This will help to find common or specific targets for the development of general control strategies centered on cytokine perception.

## Data Availability

The datasets presented in this article are available. Requests to access the datasets should be directed to Juan Jose Valdez Alarcón, jose.alarcon@umich.mx.

## Supplementary material

Supplementary Figure s1 Effect of PBS dilution of biofilm formation. *S. aureus* ATCC 27543, cells treated with different dilutions (0.025 X, 0.05 X, 0.075 X, 0.1 X) of phosphate-buffered saline (1 X PBS) or TRIS-buffered saline solution (1 X TBS). Values with different letters indicate statistically significant differences (*p* < 0.05) among treatments.

Supplementary Figure s2 Cell viability in biofilms of *S. aureus* in response to cytokines. *S. aureus* ATCC 27543. **(A)** CFU/ml of the sessile phase of the biofilm, and **(B)** CFU/ml of the planktonic phase. Different letters indicate significant differences. The number in parentheses in the graph indicates the concentration of the cytokines in ng/ml. Values with different letters indicate statistically significant differences (*p* < 0.05) among treatments.

Supplementary Figure s3 Dose – response effect of cytokines on S. aureus biofilm formation. *S. aureus* ATCC 27543 incubated with TNFα **(A)**, IL-1β **(B)** or IL-10 **(C)**. Values with different letters indicate statistically significant differences (*p* < 0.05) among treatments.

Supplementary Figure s4 CSLM of S. aureus ATCC 27543 biofilms under the effect of immunomodulatory signals. Panel **(A)** experimental controls; panel **(B)** cytokine treatments. The treatments used were TSB alone or supplemented with 0.01 X PBS, 1% glucose, 1% NaCl, IL-1β, TNF-α and IL-10. XZ profiles showing, from left to right, merged images, SYTO9 channels and PI channels. XZY profiles show only the merged images. Images are representative of at least two replicates.

Supplementary Figure s5 Thickness of S. aureus biofilms. S. aureus ATCC 27543, was stained with fluorescence and captured with the laser scanning confocal microscope and measured with the ImageJ program. **(A)**, Thickness of the biofilm as a percentage of the control. **(B)**, fractions of live (gray bars) and dead (black bars) cells of S. aureus USA 300. Plotted data are representative image areas from two experiments. The proinflammatory cytokines IL-1β, TNF-α were used at 10 and 1 ng/ml respectively; IL-10 was used 10 ng/ml. Values with different letters indicate statistically significant differences (*p* < 0.05) among treatments.
