# Supplementary figures and images for: IL-1β, TNF-α, and IL-10 reduce cell viability and differentially alter biofilm structure and gene expression levels in Staphylococcus aureus USA 300
Source: Front Immunol. 2025 Dec 5;16:1665397. doi: 10.3389/fimmu.2025.1665397 (PMC12714981; doi:10.3389/fimmu.2025.1665397)

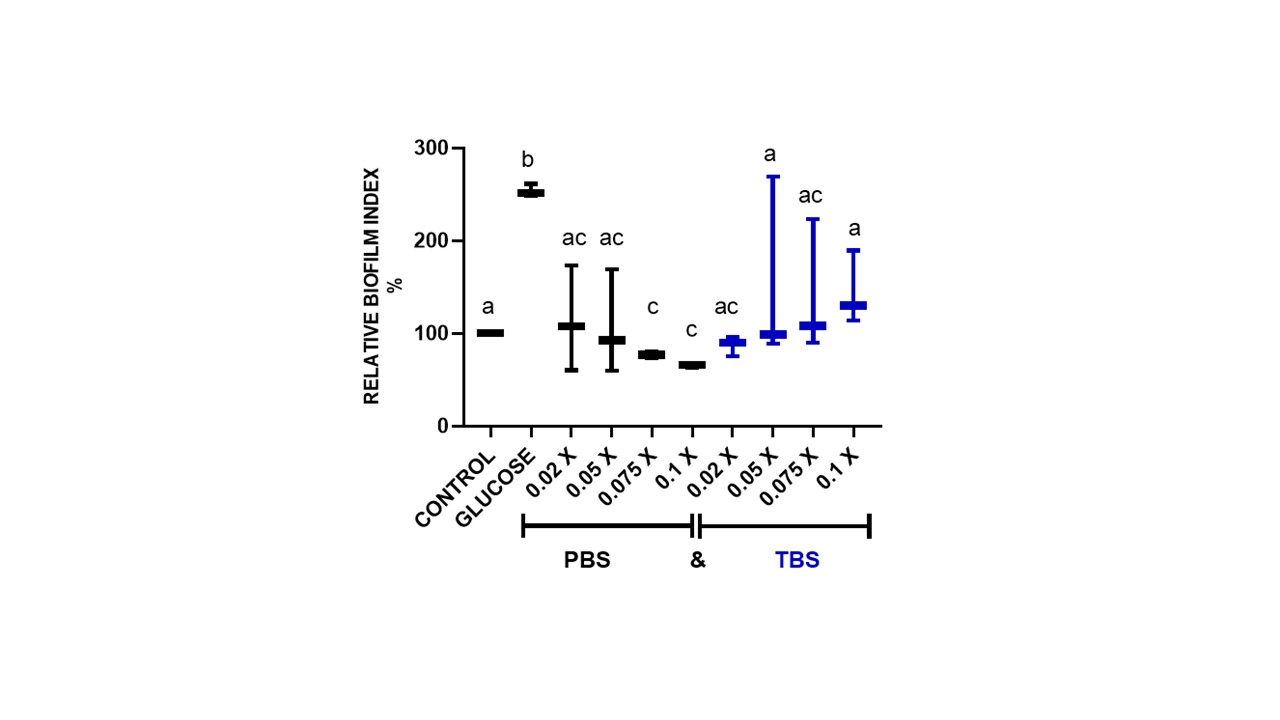

Supplement: Supplementary Figure s1 — Effect of PBS dilution of biofilm formation. S. aureus ATCC 27543, cells treated with different dilutions (0.025 X, 0.05 X, 0.075 X, 0.1 X) of phosphate-buffered saline (1 X PBS) or TRIS-buffered saline solution (1 X TBS). Values with different letters indicate statistically significant differences (p < 0.05) among treatments. [file Image1.jpeg]

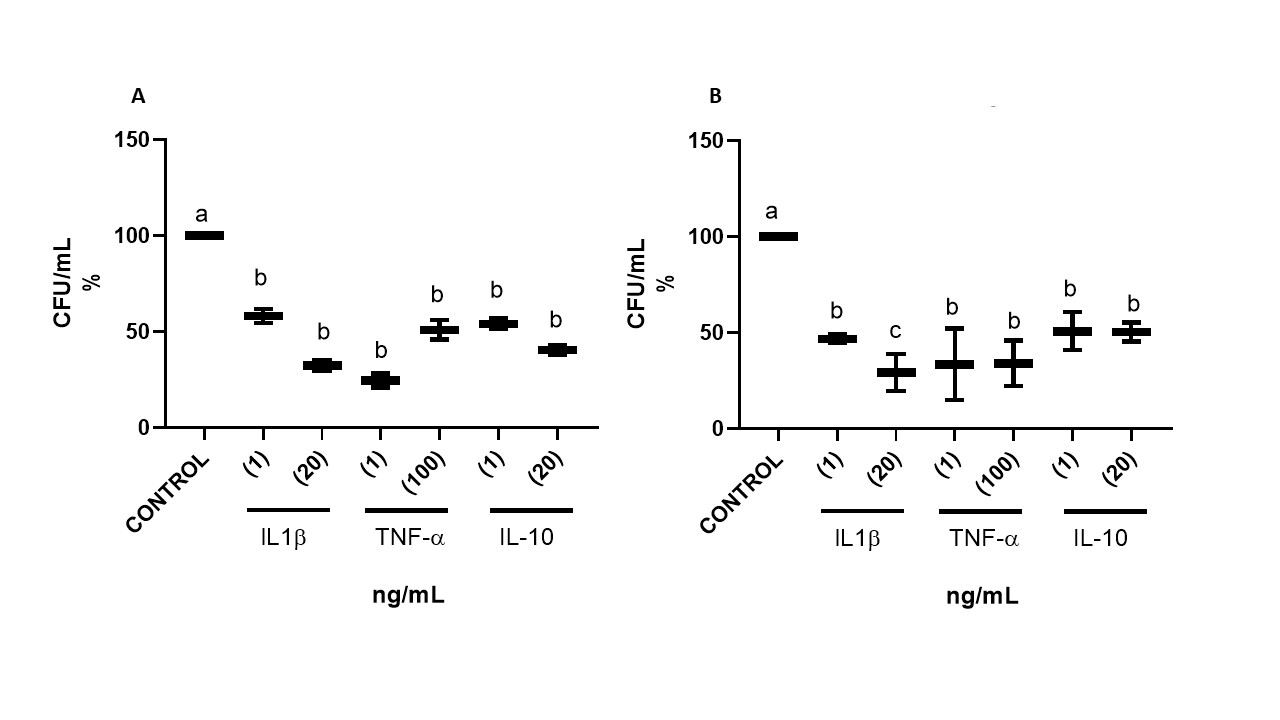

Supplement: Supplementary Figure s2 — Cell viability in biofilms of S. aureus in response to cytokines. S. aureus ATCC 27543. (A) CFU/ml of the sessile phase of the biofilm, and (B) CFU/ml of the planktonic phase. Different letters indicate significant differences. The number in parentheses in the graph indicates the concentration of the cytokines in ng/ml. Values with different letters indicate statistically significant differences (p < 0.05) among treatments. [file Image2.jpeg]

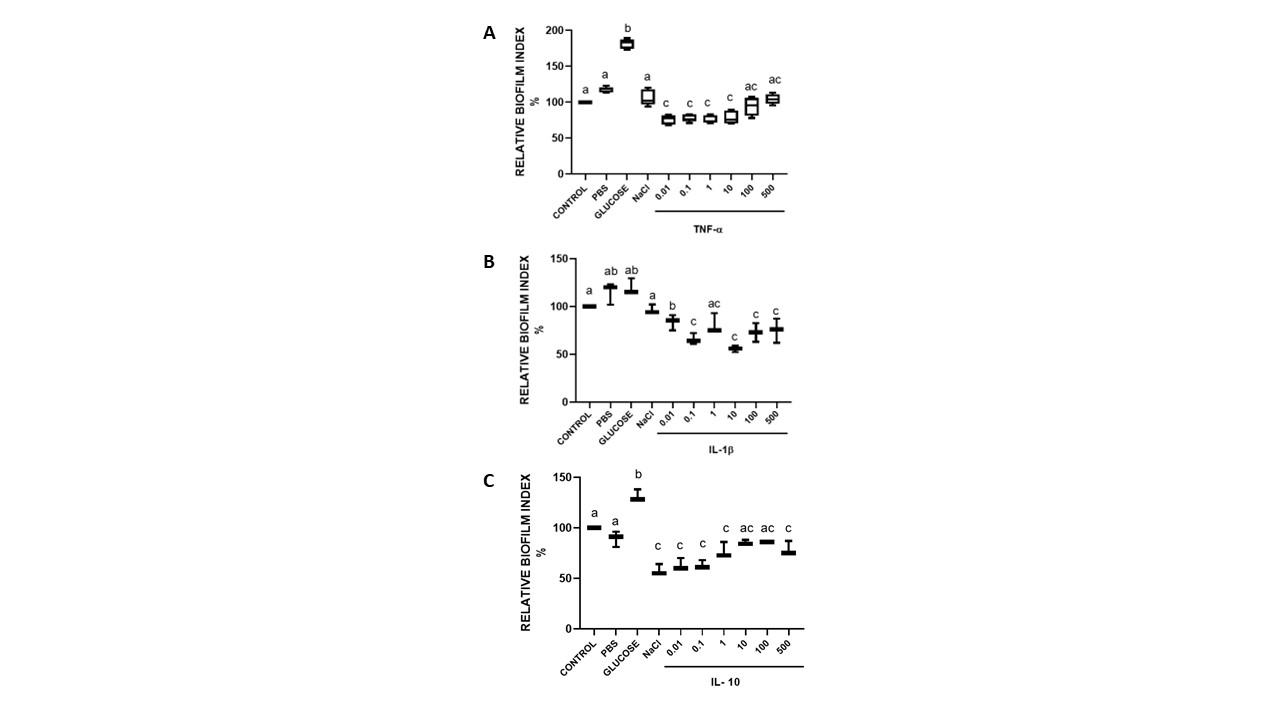

Supplement: Supplementary Figure s3 — Dose – response effect of cytokines on S. aureus biofilm formation. S. aureus ATCC 27543 incubated with TNFα (A), IL-1β (B) or IL-10 (C). Values with different letters indicate statistically significant differences (p < 0.05) among treatments. [file Image3.jpeg]

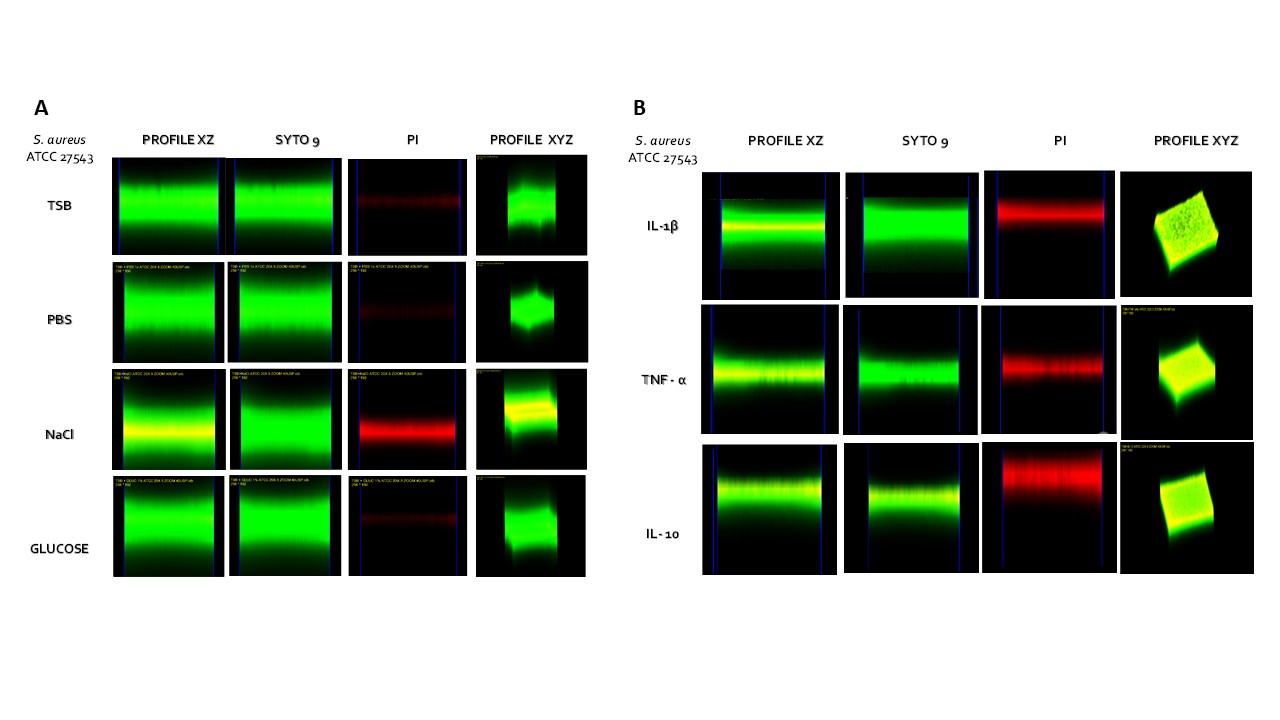

Supplement: Supplementary Figure s4 — CSLM of S. aureus ATCC 27543 biofilms under the effect of immunomodulatory signals. Panel (A) experimental controls; panel (B) cytokine treatments. The treatments used were TSB alone or supplemented with 0.01 X PBS, 1% glucose, 1% NaCl, IL-1β, TNF-α and IL-10. XZ profiles showing, from left to right, merged images, SYTO9 channels and PI channels. XZY profiles show only the merged images. Images are representative of at least two replicates. [file Image4.jpeg]

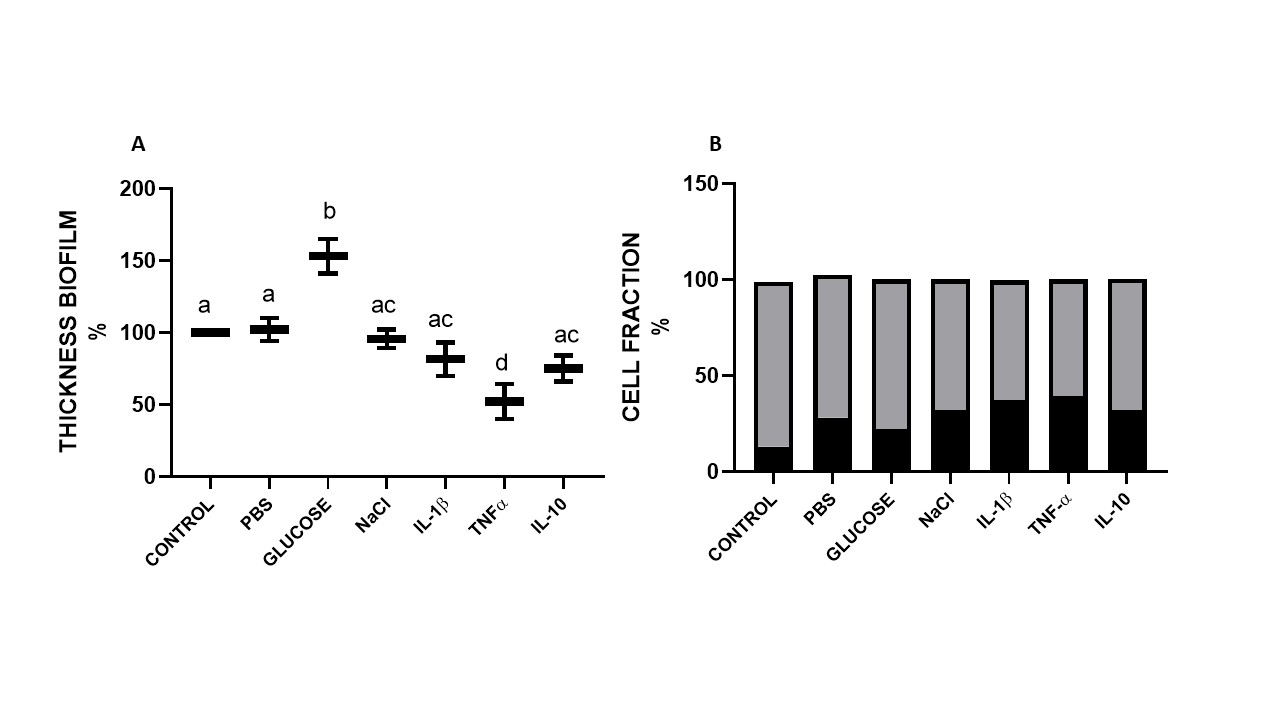

Supplement: Supplementary Figure s5 — Thickness of S. aureus biofilms. S. aureus ATCC 27543, was stained with fluorescence and captured with the laser scanning confocal microscope and measured with the ImageJ program. (A), Thickness of the biofilm as a percentage of the control. (B), fractions of live (gray bars) and dead (black bars) cells of S. aureus USA 300. Plotted data are representative image areas from two experiments. The proinflammatory cytokines IL-1β, TNF-α were used at 10 and 1 ng/ml respectively; IL-10 was used 10 ng/ml. Values with different letters indicate statistically significant differences (p < 0.05) among treatments. [file Image5.jpeg]
